# AIM2 Co-immunization with VP1 Is Associated with Increased Memory CD8 T Cells and Mounts Long Lasting Protection against Coxsackievirus B3 Challenge

**DOI:** 10.3389/fcimb.2017.00247

**Published:** 2017-06-08

**Authors:** Liang Yin, Dafei Chai, Yan Yue, Chunsheng Dong, Sidong Xiong

**Affiliations:** Jiangsu Key Laboratory of Infection and Immunity, Institutes of Biology and Medical Sciences, Soochow UniversitySuzhou, China

**Keywords:** Coxsackievirus B3, viral myocarditis, DNA vaccine, adjuvant, AIM2, memory CD8 T cells

## Abstract

The recurrent Coxsackievirus B3 (CVB3) infection is the most important cause of intractable myocarditis which often leads to chronic myocarditis and even dilated cardiomyopathy. Therefore, enhanced DNA vaccines capable of memory CD8 T cells are essential for long-lasting immunological protection against CVB3 infection. In this study, absent in melanoma 2 (AIM2) was used as an adjuvant to enhance the induction of memory CD8 T cells elicited by VP1 (viral capsid protein 1) vaccine. Mice were intramuscularly injected with 50 μg AIM2 plasmid and equal amount of VP1 plasmid (pAIM2/pVP1) vaccine 4 times at 2 week-intervals. We observed that the protection of pAIM2/pVP1 vaccine against CVB3 challenge was evidenced by significantly improved cardiac function, reduced myocardial injuries, and increased survival rate when compared with immunization with pVP1. Co-immunization with pAIM2/pVP1 robustly augmented T lymphocytes proliferation and CVB3-specific cytotoxic T lymphocyte responses. Importantly, 16 weeks after the last immunization, pAIM2/pVP1 co-immunization significantly enhanced the expression of Bcl-6, SOCS3, and Sca-1 which are critical for memory CD8 T cells as compared with pVP1 immunization. Notably, CD8 T cells that are likely vaccine-induced memory T cells were responsible for the protective efficacy of pAIM2/pVP1 vaccine by abolition of a CD8 T cell immune response following a lethal dose of CVB3 infection. Our results indicate that AIM2-adjuvanted vaccine could be a potential and promising approach to promote a long-lasting protection against CVB3-induced myocarditis.

## Introduction

Coxsackievirus B3 (CVB3) is one of non-enveloped, positive-sense, single-stranded RNA viruses and it is a principle etiologic agent in myocarditis (Palmenberg, 1990; Tam, 2006; Bailey and Tapprich, 2007). CVB3 infection can induce an acute or chronic viral myocarditis and ultimately lead to dilated cardiomyopathy (Kawai, 1999; Feldman and McNamara, 2000). Various of vaccines against CVB3 have been examined in animal models, including inactivated or attenuated virus vaccines, DNA vaccines, recombinant protein vaccines, and virus-like particle vaccines (Li et al., 2016). However, there are no efficient vaccines or therapeutic reagents available for a long-lasting protection against CVB3 infection (Fairweather et al., 2004; Xu et al., 2004; Chen et al., 2011; Guglin and Nallamshetty, 2012). Therefore, there is an obvious need to develop new and efficient vaccines for the long-term protection against CVB3 infection.

DNA vaccination is an attractive and safe approach to generate protective immune response. However, compared with other candidates, the relatively low efficacy of DNA vaccines are in inducing protective immune responses which has impaired their practical use, especially in humans and other large animal species (Scheerlinck, 2001). Discovery of safe effective adjuvants might be valuable to a successful DNA vaccine. Memory CD8 T cell immune responses are crucial for the long-lasting anti-CVB3 protection (Wherry and Ahmed, 2004; Kemball et al., 2008). Vaccination strategies are able to induce potent memory T cells immune responses that are likely to be the most successful in preventing the subsequent CVB3 infection and progression to chronic myocarditis or dilated cardiomyopathy (Henke et al., 2001; Maisch and Pankuweit, 2012; Griffiths and Khader, 2014). Thus, the adjuvant of VP1 antigen is important for the generation of memory immunity. In our previous study, DNA sensor AIM2 could act as a mucosal adjuvant for VP1 vaccine to induce mucosal immune responses against CVB3 challenge (Chai et al., 2014). Recently, study found that the humoral and cellular antigen-specific adaptive responses were both reduced in AIM2^−/−^ mice after DNA vaccination (Suschak et al., 2015). A more recent study showed that Mycobacterium bovis BCG heterologously expressing the esx-1 region of Mycobacterium marinum induces the cGAS/STING/TBK1/IRF-3/type I interferon axis and enhances AIM2 and NLRP3 inflammasome activity, resulting in both higher proportions of CD8 T cell effectors against mycobacterial antigens and polyfunctional CD4^+^ Th1 cells, further suggesting the role of AIM2 as a sensor for DNA vaccines. However, whether AIM2 enhance memory CD8 T cell immune responses by VP1 intramuscular vaccine remains unclear.

In the present work, we reported intramuscular co-immunization of pAIM2/pVP1 effectively prevented the CVB3-induced myocarditis 16 weeks post vaccination. A significantly higher frequency and number of memory CD8 T cells in pAIM2/pVP1 co-immunization compared to pVP1 immunization. We further examined SOCS3, Bcl-6, Sca-1, T-bet, and Blimp-1 genes, which are associated with memory T cell differentiation, were increased with pAIM2/pVP1 co-immunization. CD8 T cell depletion significantly increased the disease severity in pAIM2/pVP1 immunized mice. Thus, intramuscular vaccination with pAIM2/pVP1 is able to induce long-lasting immune responses against viral myocarditis, which may owe to enhanced memory CD8 T cell response.

## Materials and methods

### Mice

Male BALB/c (H-2^d^) mice, 6-week-old, were purchased from the Experimental Animal Center of Chinese Academy of Science (Shanghai, China). All Mice were bred and maintained under pathogen-free conditions, and all animal experiments were performed according to the guidelines for the Care and Use of Laboratory Animals (Ministry of Health, China, 1998). All animal experiments were performed in accordance with the laboratory animals' guidelines of the Laboratory Animal Ethical Commission of Soochow University (SYXK2014-0030).

### Cells and virus

The human cervical adenocarcinoma cell line HeLa (ATCC number: CCL-2) was cultured in RPMI medium 1640 (Gibco, USA) supplemented with 10% heat-inactivated fetal bovine serum (FBS; Gibco, USA), 100 U/ml penicillin and 100 μg/ml streptomycin at 37°C in a 5%CO_2_ incubator. The Nancy strain CVB3 was a gift from Professor Yingzhen Yang (Key Laboratory of Viral Heart Diseases, Zhongshan Hospital, Shanghai Medical college of Fudan University) and was maintained in HeLa cells with 2% FBS and used for viral challenge assay after three passages. Viral titer was routinely determined prior to infection by a 50% lethal dose (LD_50_) assay according to previously published procedures (Henke et al., 1995).

### Immunization protocol

The mice were randomly divided into four groups: pCDNA3.1 vector control (mock), pEF-BOS-AIM2 (pAIM2, murine AIM2: NM_001013779.2), pcDNA3.1-VP1 (pVP1, VP1: M33854.1), and pEF-BOS-AIM2 plus pcDNA3.1-VP1 (pAIM2/pVP1). For pAIM2/pVP1 induced immune response and protection detection, each group consisted of 8 mice. For the mice survival rate detection, each group consisted of 12 mice. The mice were injected with 30 μl of 0.25% bupivacaine into the quadricep of each hind leg 3 d before DNA immunization. Plasmid DNA pAIM2/pVP1 (each 50 μg) was intramuscularly injected into the same area and immunization was 4 times at 2-week intervals. For pVP1, pAIM2 or mock immunized group, mice were received additional 50 μg pcDNA3.1 vector to make sure that the total DNA amount was 100 μg.

### Flow cytometry analysis

For cytokine assay, splenocytes were distributed in a 24-well culture plate at a density of 1 × 10^6^/ml and stimulated with 10 ug/ml VP1 protein for 72 h. These cells were also stimulated with 500 ng/ml Ionomycin (Sigma-Aldrich) and 50 ng/ml PMA (Sigma-Aldrich) plus 5 ng/ml Brefeldin A (eBioscience) at 37°C and 5% CO_2_ for the last 5 h. And then splenocytes were collected and stained with the following anti-mouse antibodies: PE-conjugated IFN-γ^+^, APC-conjugated CD8 (dilution 1:100, Biolegend, San Diego, CA). For detection of memory CD8 T cells, splenocytes from immunized mice were isolated and performed cell surface staining with the following anti-mouse antibodies: APC-conjugated CD8, FITC-conjugated CD44, and PE-conjugated CD62L (dilution 1:100, Biolegend, San Diego, CA). All flow cytometry data were acquired on a BD FACSCanto II (BD Biosciences) in FACSDiva software (BD Biosciences) and analyzed by FlowJo software (Tree Star Inc.).

### CD8 T cell purification

Single cell suspension of spleen cells from immunized mice was prepared. CD8 T cells were purified (about 90% purity) from spleen cells by magnetic beads (CD8 T cell isolation kit, MACS beads; Miltenyi Biotec) according to the protocol provided by the manufacturer.

### Isolation of RNA and real-time RT-PCR

Total RNA was isolated from purified CD8 T cells using TRIzol reagent (Invitrogen). cDNA was prepared with a cDNA synthesis kit (Takara) according to the manufacturer's instructions. Transcript levels were determined by quantitative real-time PCR using SYBR Green PCR Master Mix (Invitrogen) on the realplex Mastercycler (Eppendorf) according to the manufacturer's instructions. The relative amount of mRNA was calculated by plotting the Ct (cycle number) and the average relative expression of genes for each group was determined using the comparative method (2^−ΔΔCt^). Primers were used as follows: GAPDH, 5′-GAG CCA AAC GGG TCA TCA TCT-3′ (forward) and 5′-GAG GGG CCA TCC ACA GTC TT-3′ (reverse); SOCS3: 5′-GAG ATT TCG CTT CGG GAC TA-3′ (forward) and 5′-ACT TGC TGT GGG TGA CCA T -3′ (reverse); Bcl-6: 5′-GTG AGC CGT GAG CAG TTT AG-3′ (forward) and 5′-CTC AGG GCT GAT TTC AGG AT -3′ (reverse); Sca-1: 5′-TGG ATT CTC AAA CAA GGA AAG TAA AGA-3′ (forward) and 5′-ACC CAG GAT CTC CAT ACT TTC AAT A-3′ (reverse); Eomes: 5′-AAG CGG ACA ATA ACA TGC AG-3′ (forward) and 5′-TGT TGT TGT TTG CAC CTT TG-3′ (reverse); T-bet: 5′-CTG CCT GCA GTG CTT CTA AC-3′ (forward) and 5′-AAG TTC TCC CGG AAT CCT TT-3′ (reverse); Blimp1: 5′-CAA GCC GAG GCA TCC TTA-3′ (forward) and 5′-CGT GTT CCC TTC GGT ATG TA-3′ (reverse).

### CVB3 infection and evaluation of viral myocarditis

Mice were intraperitoneally infected with 3 × 50% lethal dose (LD_50_) CVB3. Seven days later, hearts were collected and fixed in 10% phosphate-buffered formalin, paraffin embedded, sectioned and stained with hematoxylin and eosin (HE). For survival rate, a lethal dose of CVB3 (5LD_50_) was administrated and survival rate was monitored until day 28.

### Anesthesia and echocardiography

Mice underwent echocardiographic measurements before sacrifice on day 7 post CVB3 infection. The mouse was positioned on a heating pad to maintain normothermia. Isoflurane was administered by a Vevo compact anesthesia system (VisualSonics, Toronto, Canada). Isoflurane induction was performed by the same individual, with the nose of the mouse in a small nose cone and with 2% isoflurane in pure medical oxygen. Assessment of cardiac function of mice was performed using high-resolution ultrasound imaging system (Vevo2100, Visual Sonics, Toronto, Canada) equipped with a 30-MHz microscan transducer. The echocardiographic measurements of left ventricular ejection fraction (LVEF) and left ventricular fractional shortening (LVFS) were performed according to the operator's manual.

### Measurement of CK and CK-MB

Serum creatine kinase (CK) and creatine kinase MB isoenzyme (CK-MB) were detected by Suzhou Kowloon Hospital (Suzhou, China).

### CD8 T cell depletions

For CD8 T cell depletions, 250 μg CD8 mAb (250 μl total volume) was administered i.p. 3 d and 1 d before CVB3 infection, which resulted in depletion of ≥90% of CD8 T cells (Steitz et al., 2001; Yauch et al., 2010; Van De Voort et al., 2013).

### Cytotoxic T lymphocyte (CTL) assays

Splenocytes were cultured in RPMI 1640 medium containing 50 U/ml IL-2 and 10 ug/ml VP1 protein for 7 d at 37°C 5% CO_2_. The processed splenocytes were washed and resuspended as effector cells in RPMI 1640. VP1-transfected autologous SP2/0 (H-2 d) cells were used as target cells. Briefly, effector cells and target cells were titrated in U-bottom 96-well plates with 50:1, 25:1, and 12.5:1 ratios of effector-target cell. Afterward, 1 × 10^4^ target cells were added and incubated at 37°C for 72 h. Cytotoxicity was determined by measuring the amount of lactate dehydrogenase (LDH) in the supernatant with the Cytotoxicity Detection Kit PLUS (LDH) (Roche).

### Histomorphological analysis

Heart tissues were fixed in 10% phosphate-buffered formalin, paraffin embedded, sectioned and stained with hematoxylin and eosin (HE). The histopathological changes of immunized mice were compared quantitatively by calculating the histopathological scores (Rezkalla et al., 1988): 0, no lesions; 1, lesions involving <25%; 2, lesions involving 25–50%; 3, lesions involving 50–75%; 4, lesions involving >75%. Two independent researchers scored separately in a blinded manner.

### Quantization of viral burden in heart

The viral burden was evaluated in heart tissues after CVB3 challenge, heart tissues were collected, weighed and frozen at −70°C in RPMI 1640 containing 10% FBS. Samples were later thawed, homogenized, serially diluted in 10-fold increments, and incubated on confluent HeLa cells monolayer for 1 h at 37°C and 5% CO_2_ to allow viral attachment, and then incubated for 7 days to allow plaque formation. Virus titers were expressed as the mean plaque forming unit (PFU)/100 mg tissue ± SD.

### Statistical analysis

Statistical analyses were performed with GraphPad Prism 5.01 Software (La Jolla, CA) statistical program. All data were given as mean and standard deviation. The data were statistically analyzed by using One-Way ANOVA followed by Tukey's *post-hoc* test. The statistical significance between pVP1 and pVP1/pAIM2 groups was indicated and set to *p* < 0.05.

## Results

### pAIM2/pVP1 co-immunization provides a long-lasting protection against CVB3-induced myocarditis

**Figure 1:**
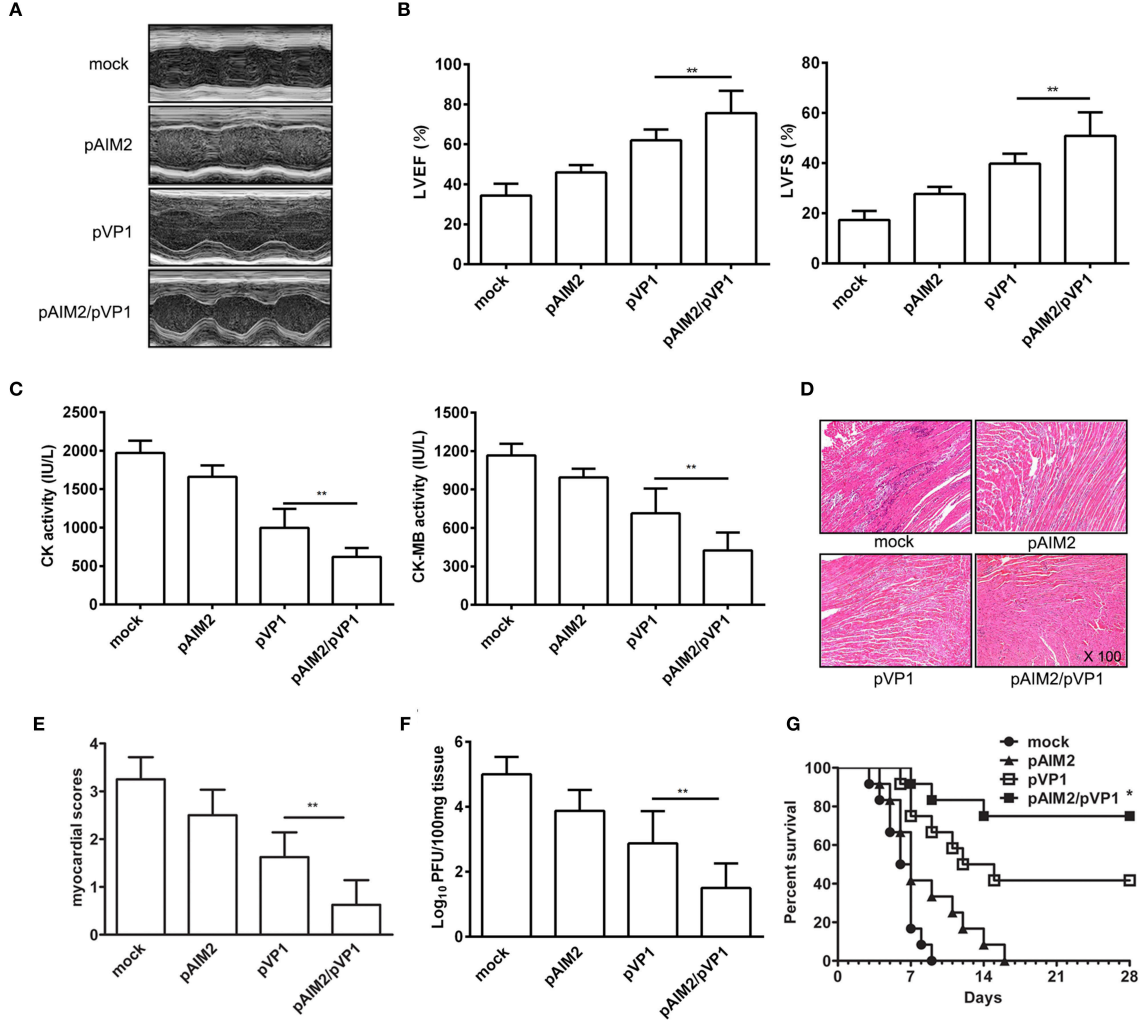
The long-lasting resistance to CVB3-induced acute myocarditis by pAIM2/pVP1 co-immunization. Sixteen weeks after the last immunization, mice were infected with 3LD_50_ CVB3 and the protect efficacy was evaluated 7 days after challenge. **(A)** Representative M-mode echocardiograms. **(B)** LVFS and LVEF from chocardiographic data. **(C)** Serum CK, CK-MB activity. **(D)** The HE staining of representative heart section (magnification: 100×). **(E)** Myocardial histopathological scores. **(F)** Viral titers. **(G)** The survival rate of mice (*n* = 12) was observed until day 28 following a lethal dose of CVB3 (5LD_50_) infection. Data are from one experiment of three independent experiments and presented as the mean ± SD. ^*^p < 0.05, ^**^p < 0.01.

To explore the long-lasting protection efficacy of pAIM2/pVP1 vaccine, 16 weeks after the last immunization, groups of mice were intraperitoneally infected with a normal lethal dose of CVB3 (3LD_50_/mouse) for the induction of acute myocarditis. Seven days post-infection, the disease severity of CVB3-induced myocarditis was evaluated. As shown in Figures 1A,B, the echocardiographic measurements demonstrated that the pAIM2/pVP1 co-immunization significantly improved the cardiac function reflected by left ventricular ejection fraction (LVEF) and left ventricular fractional shortening (LVFS) as compared with pVP1 immunized group. Consistently, the myocardial injury reflected by the serological indexes of CK and CK-MB levels was significantly lower in pAIM2/pVP1 immunized mice than those in pVP1 immunized mice (Figure 1C). Histological analysis of HE-stained heart sections showed that tiny areas of myocytes necrosis and infiltrating inflammatory cells were observed in pAIM2/pVP1 co-immunization group (Figure 1D). The myocardial pathology score was also significantly reduced in pAIM2/pVP1 immunized mice compared with pVP1 immunized mice (Figure 1E). More importantly, the virus load was decreased in heart tissue from pAIM2/pVP1 immunized mice compared with those from pVP1 immunized mice (Figure 1F), indicating pAIM2/pVP1 immunization results in more efficient viral cleaning. To further confirm the improved immunoprotection conferred by pAIM2/pVP1 co-immunization, mice were challenged with a lethal dose of CVB3 (5LD_50_) and survival rate was observed up to 28 days. As shown in Figure 1G, all of the mock immunized mice died within 9 days of CVB3 challenge, while about 40% of the mice in pVP1 immunization group survived from the lethal challenge (*P* < 0.05). An increased survival rate (about 75%) was observed in pAIM2/pVP1 co-immunization group. These results suggest that pAIM2/pVP1 co-immunization can produce a long-lasting protection against CVB3-induced myocarditis.

### pAIM2/pVP1 co-immunization augments CD8 T cell immune response

**Figure 2:**
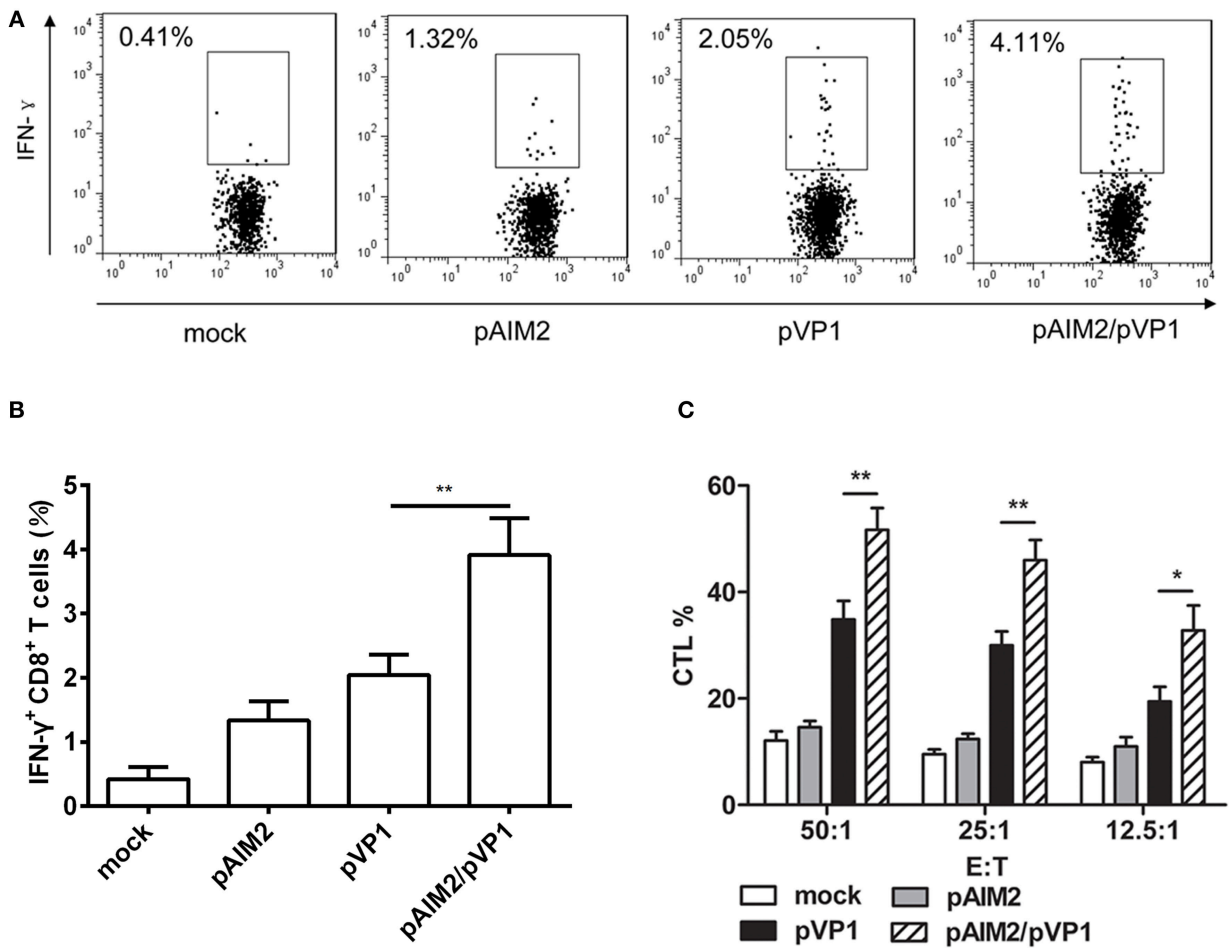
CVB3-specific CTL activity elicited by pAIM2/pVP1 vaccine. Spleen cells from immunized mice (*n* = 8) were harvested and stimulated *in vitro*. **(A,B)** Flow cytometry analyses were performed on spleen cells to assess percentages of IFN-γ^+^CD8^+^ T cells. **(C)** Cytotoxic T lymphocyte (CTL) activity in spleen cells. Data are from one experiment of three independent experiments and presented as the mean ± SD. ^*^p < 0.05, ^**^p < 0.01.

Given the important role of CD8 T cells in the defense of viral infection by inducing cytotoxicity or through promoting cytokines such as IFN-γ, we assess the CD8 T cell-mediated immune responses in immunization group without CVB3 infection. Intracellular staining results showed that the percentage of IFN-γ secreting CD8^+^ T cells in pAIM2/pVP1 co-immunization group was significantly higher than in pVP1 immunized group (Figures 2A,B). Compared with pVP1 immunized group, CVB3-specific CTL activity was remarkably enhanced in pAIM2/pVP1 co-immunization group (Figure 2C). These results showed that pAIM2/pVP1 vaccination induced robust specific T cell immune response and protected mice from CVB3 infection 16 weeks post vaccination.

### pAIM2/pVP1 co-immunization promoted expression of specific transcription factors and induction of memory CD8 T cells

**Figure 3:**
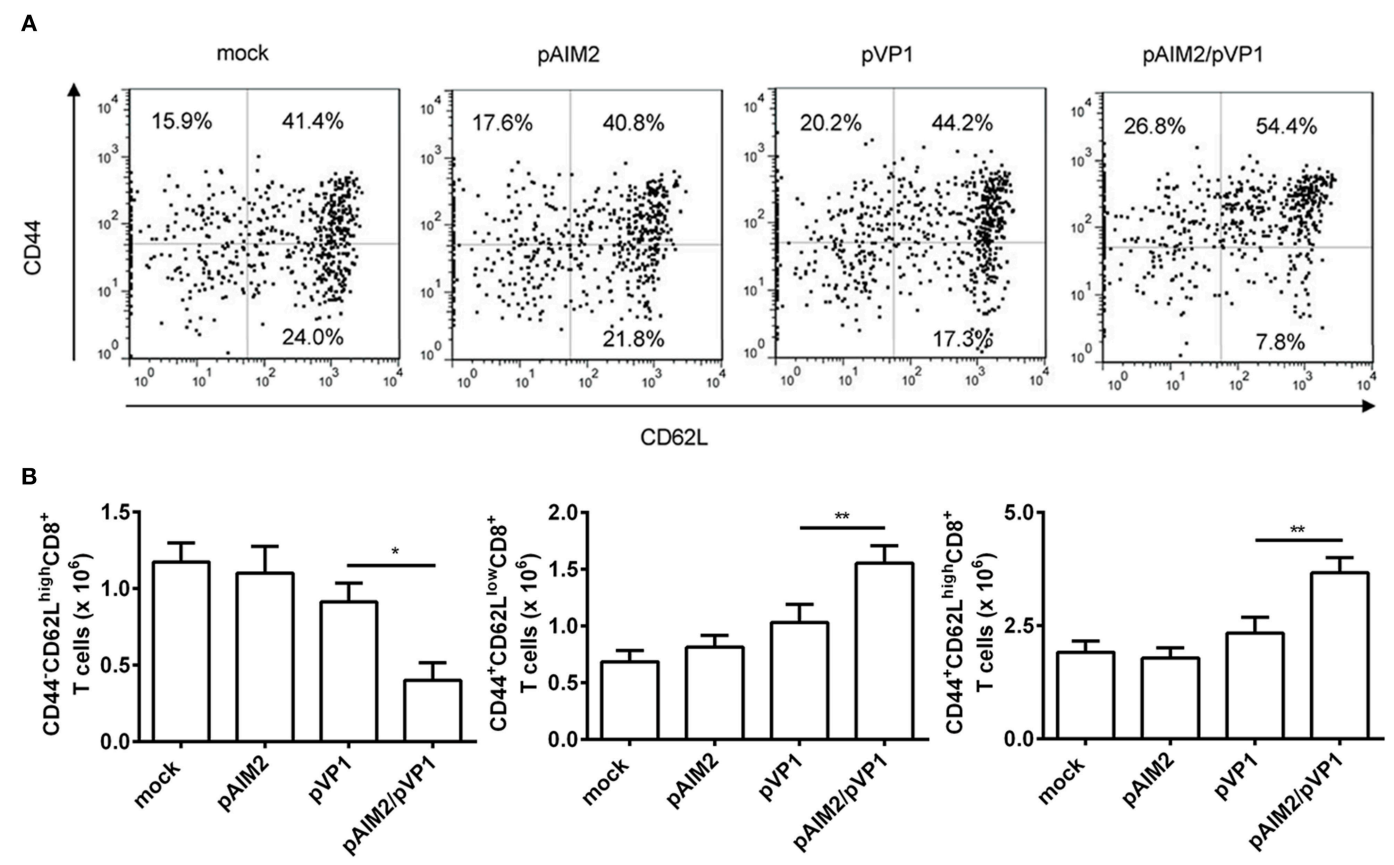
The enhancement of memory CD8 T cells by pAIM2/pVP1 co-immunization. 16 weeks after the last immunization, spleen cells from each immunization groups (*n* = 8 per group) were harvested and analyzed by immunofluorescence staining and flow cytometry. **(A)** The frequency of CD8 T cells showing memory subset phenotypes: naive cells (CD44^−^CD62L^high^), effector memory (CD44^+^CD62L^low^), and central memory (CD44^+^CD62L^high^) in spleen from various vaccine immunized mice. One representative flow cytometry result was shown for per group. **(B)** Statistical analysis of the absolute number of naive, effector memory, and central memory cells in spleen. Data are from one experiment of three independent experiments and presented as the mean ± SD. ^*^p < 0.05, ^**^p < 0.01.

Memory CD8 T cells play a central, initiating role in recall immune responses to infection (Singh et al., 2010). CD44 is a surface protein required for lymphocyte extravasation to inflammatory sites and the increasement of CD44 represents a marker for memory T cells (Harty et al., 2000). CD62L is a lymph node homing receptor that is decreased upon CTL activation (Zhang et al., 2017). Therefore, the induction of different CD8 memory T cell subsets was determined by CD44 and CD62L staining 16 weeks after the final immunization. The percentage and number of effector memory (CD44^+^CD62L^low^) and central memory (CD44^+^CD62L^high^) CD8 T cells were significantly increased in the spleen of mice co-immunized with pAIM2/pVP1 as compared with mice immunized with pVP1 alone, whereas we observed that the naive CD8^+^ T cells (CD44^−^CD62L^high^) were decreased (Figures 3A,B). These data indicated that pAIM2/pVP1 co-immunization were crucial for the induction of memory CD8 T cells in the absence of CVB3 infection, which may facilitate to provide a longer-lasting protection against CVB3 challenge.

**Figure 4:**
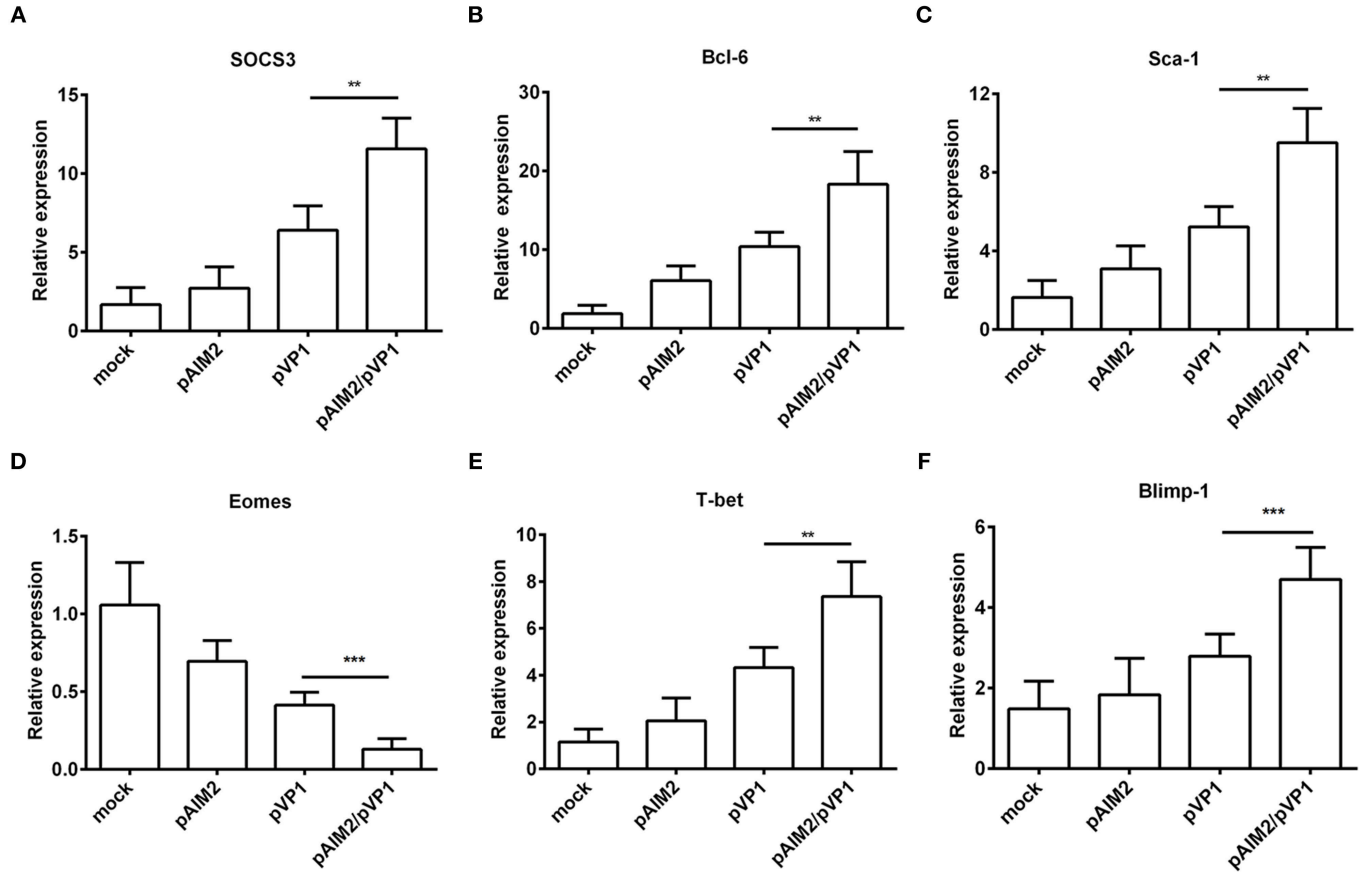
The expression of memory transcription factors was increased in CD8^+^ T cells. The mRNA levels of transcription factors **(A)** SOCS3, **(B)** Bcl-6, **(C)** Sca-1, **(D)** Eomes, **(E)** T-bet and **(F)** Blimp-1 associated with memory CD8 T cells differentiation were analyzed in CD8 T cells of various vaccines immunized mice (*n* = 8 per group) by real-time PCR. Data are from one experiment of three independent experiments and presented as the mean ± SD. ^**^p < 0.01, ^***^p < 0.001.

To further understand the mechanisms of AIM2-mediated effects on the induction of memory CD8 T cells, we examined the expression of specific transcription factors for differentiation of memory CD8 T cells at 16 weeks after the last immunization without CVB3 infection. As shown in Figures 4A–F significant increasement of SOCS3, Bcl-6, Sca-1, T-bet, and Blimp-1 genes was observed in CD8^+^ T cells from spleen of pAIM2/pVP1 immunized mice as compared with pVP1 immunized mice. However, expression of Eomes gene was reduced in CD8 T cells from spleen of mice immunized with pAIM2/pVP1. Thus, these data indicate that pAIM2/pVP1 co-immunization might be closely related with the induction of memory CD8 T cells through these specific transcription factors.

### CD8 T cells are associated with the protective efficacy of pAIM2/pVP1 co-immunization

**Figure 5:**
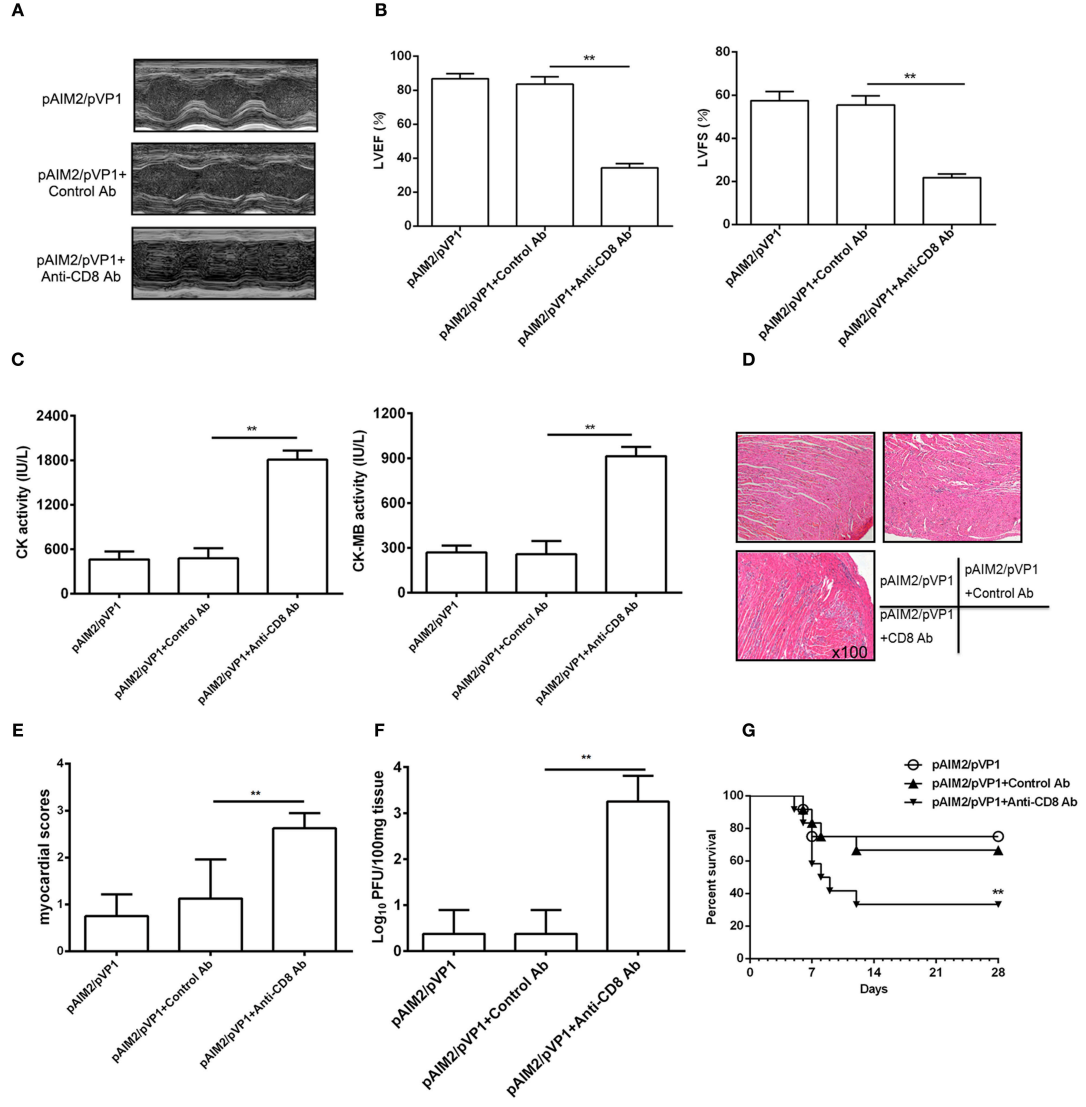
CD8 T cells are associated with the protective efficacy of pAIM2/pVP1 vaccine. BALB/c mice (*n* = 8 per group) immunized with pAIM2/pVP1 were injected with CD8 or control antibodies at -3 d and -1 d following CVB3 infection. Seven days after CVB3 challenge, the protection efficacy was evaluated in recipient mice. **(A)** Representative M-mode echocardiograms. **(B)** LVFS and LVEF from chocardiographic data. **(C)** Serum CK, CK-MB activity. **(D)** The representative heart section (magnification: 100×). **(E)** Myocardial histopathological scores. **(F)** Viral titers in the heart tissues. **(G)** The survival rate of mice (*n* = 12 per group) was observed until day 28 following a lethal dose of CVB3 (5LD_50_) infection. Data are from one experiment of three independent experiments and presented as the mean ± SD. ^**^p < 0.01.

To address the role of memory CD8 T cells during CVB3 challenge, we deleted CD8 T cells by administrating mice with CD8 mAb 16 weeks post pAIM2/pVP1 immunization. Seven days after CVB3 infection (3LD_50_/mouse), the protection efficacy of myocarditis was significantly declined in mice group with CD8 mAb treatment verified by reduced LVEF and LVFS (Figures 5A,B), increased myocardial injury (Figure 5C), myocardial pathology (Figures 5D,E), and virus load (Figure 5F). Mice were also challenged with 5LD_50_ of CVB3 and the pAIM2/pVP1 immunized mice with CD8 antibody treatment showed the significantly decreased survival rate compared with that of control mice (Figure 5G), indicating that CD8 T cells that are likely vaccine-induced memory T cells are responsible for the protective efficacy of pAIM2/pVP1 vaccine against CVB3 challenge.

## Discussion

CVB3 infection could induce acute myocarditis and proceed to a chronic phase, and ultimately lead to dilated cardiomyopathy (Kawai, 1999; Feldman and McNamara, 2000). Several studies demonstrate that DNA vaccination is an attractive approach to induce long-lasting T cells immunity against virus infection and reduce virus-associated myocardial dysfunction (Kim et al., 2004; Hansen et al., 2011; Rigato et al., 2011; Jalah et al., 2012). The potent adjuvants and vaccination modalities are needed for successful of DNA vaccines to induce memory T cells immune responses (Esser et al., 2003; MacLeod et al., 2011). A variety of hypothetical safety concern include the possibility of an increased risk of autoimmune disease has become the key reason of the adoption of new adjuvants into licensed vaccines. AIM2 is a host protein and vaccination with pAIM2 might have autoimmunity risk. We intramuscularly injected the pAIM2 plasmid and it supposes to be intracellular expressed and presented with MHC I to particularly active cellular immune response. In this study, we didn't observe autoimmunity disease occurred in the immunized mice indicating that the autoimmunity caused by pAIM2 vaccination is minor.

Pattern recognition receptors (PRRs) recognizing intracellular DNA could potentially be used to enhance intrinsic immune-stimulating properties of plasmid DNA vaccines (Kumar et al., 2009; Kumagai and Akira, 2011). The study demonstrates that DAI boosts DNA-sensing innate immune activation and thereby generates the effective CTL induction and long-lasting antitumor immunity (Lladser et al., 2011). Cytosolic DNA sensor AIM2 as a member of PRRs has a PYRIN domain at its N-terminus and a HIN-200 domain directly involved in dsDNA recognition at its C-terminus (Roberts et al., 2009; Choubey et al., 2010). AIM2 binds to dsDNA and then forms inflammasome to activate the procaspase-1. The cleavage of pro-interleukin-1β by caspase-1 results in the secretion of interleukin-1β (IL-1β) (Fernandes-Alnemri et al., 2009, 2010; Hornung et al., 2009; Schroder et al., 2009; Jones et al., 2010; Rathinam et al., 2010), which could recruit immune cells and regulate immune-cell proliferation and differentiation (Dinarello, 2009; Sims and Smith, 2010). Previous study reports AIM2 inflammasome as a DNA vaccine sensor was able to regulate the antigen-specific adaptive immune response. Importantly, the efficacy of DNA vaccination was independent on the production of IL-1β and IL-18. AIM2 plasmids could augment the production of IFN-α/β by surrounding cells, possibly through the STING/TBK1-IRF3 axis, further promoting the immune response. AIM2 could augment the production of IFN-α/β at the immunization site (Suschak et al., 2015). In our previous study, we have demonstrated that AIM2 as a mucosal adjuvant could promote the SIgA production and enhance the mucosal immune responses by intranasal way. CS-pAIM2/CS-pVP1 vaccine alleviate the CVB3-induced acute myocarditis (Chai et al., 2014). We also have demonstrated that CS-pAIM2/CS-pVP1 vaccine alleviate the CVB3-induced chronic myocarditis by enhancing the immunogenicity of immunogenic antigens and efficiently inducing mucosal T cell immune responses with intranasal immunization (Chai et al., 2015). In the present study, the results show that AIM2 co-immunization with VP1 could mount long-lasting immune responses against acute viral myocarditis.

Memory CD8 T cells contribute to host defenses during a wide range of viral and intracellular bacterial infections (Harty et al., 2000). Previous study reported that frequency of HBV-specific CD8 T cells induced by DNA immunization was at the peak of the primary T cell response at 10 days after DNA immunization and then slowly contracted by intramuscular injection (Furuichi et al., 2005). 90% of the Ag-specific T cells die via apoptosis at 16 weeks after last immunization and the rest cells were mainly Ag-specific memory T cells (Zheng et al., 1995; Lin et al., 2000). Therefore, we believe that, at the time point 16 weeks post pAIM2/pVP1 co-immunization, depletion of CD8 T cells abrogated enhanced protection in immunized mice. This likely reflects activity of vaccine-induced memory CD8 T cells, but potential contributions of other CD8 T cells cannot be fully ruled out. Although we detected much higher percentage of CD44^+^CD62L^Low^ and CD44^+^CD62L^high^ memory T cells following pAIM2/pVP1 immunization relative to those of pVP1-immunized mice, we were unable to determine whether these memory T cells were generated from pAIM2/pVP1 induction without antigen-specific tetramer staining. Prior study has shown that the induction of memory CD8 T cell phenotype was characterized by increasing of SOCS3, Bcl-6, and Sca-1, through STAT-3 signal (Liu et al., 2013). Therefore, we considered that AIM2 might facilitate the induction of memory CD8 T cells by enhanced transcription factors SOCS3, Bcl-6, Sca-1, T-bet, and Blimp-1 expression in our study. However, AIM2 may have more profound roles in the expansion and survival of effector CD8 T cells and persistence of memory cells in CVB3 infection. Memory CD4 T cells are also a critical component of protective immunity to infection disease (Swain et al., 2004; Zaph et al., 2006). Our experiments do not formally exclude contributions of CD4 T cells or non-memory CD8 T cells to protection in vaccinated mice, but the data support the likely possibility that vaccine-induced memory CD8 T cells protect against CVB3-induced myocarditis.

In conclusion, our results suggest that AIM2 boosts DNA-sensing innate immune activation and thereby favors the induction of memory CD8 T cells. Thus, usage of AIM2 as an adjuvant could enhance the immunogenicity of antigens efficiently to induce memory T cells immunity and have preventive potential for virus induced diseases.

## Author contributions

YY, CD, and SX: Designed the study. LY and DC: Performed the experiments. LY, DC, CD, and SX: Interpreted the data. LY, DC, CD, and SX: Wrote the manuscript. All authors approved the final version of the paper.

### Conflict of interest statement

The authors declare that the research was conducted in the absence of any commercial or financial relationships that could be construed as a potential conflict of interest.
